# A Simple Cost‐Effective Method to Fabricate Single Nanochannels by Embedding Electrospun Polyethylene Oxide Nanofibers

**DOI:** 10.1002/open.202400008

**Published:** 2024-03-21

**Authors:** Lei Zhou, Zhuonan Chen, Jian Ma

**Affiliations:** ^1^ College of Communication and Art Design University of Shanghai for Science and Technology Shanghai 200093 China; ^2^ School of Intelligent Emergency Management University of Shanghai for Science and Technology Yangpu District, Shanghai 200093 China; ^3^ Jiangsu Key Laboratory for Design and Manufacture of Micro-Nano Biomedical Instruments Nanjing 211189 China; ^4^ Engineering Research Center of New Light Sources Technology and Equipment Ministry of Education Nanjing 211189 China; ^5^ School of Mechanical Engineering Southeast University Nanjing 211189 China

**Keywords:** polyethylene oxide nanofiber, electrospinning, PDMS nanochannel, power generation

## Abstract

Solid state nanochannels provide significant practical advantages in many fields due to their interesting properties, such as controllable shape and size, robustness, ion selectivity. But their complex preparation processes severely limit their application. In this study, a simple cost‐effective method to fabricate single nanochannel by embedding a single polyethylene oxide (PEO) nanofiber is presented. Firstly, PEO nanofibers are prepared by electrospinning, and then a single PEO nanofiber are precisely transferred to the target sample using a micromanipulation platform. Then, PDMS is used for embedding, and finally, the PEO nanofiber is dissolved to obtain a single nanochannel. Unlike other methods of preparing nanochannels by embedding nanofibers, this method can prepare single nanochannel. The diameter of nanochannel can be as fine as 100 nm, and the length can reach several micrometers. The power generation between two potassium chloride solutions with various combinations of concentrations was investigated using the nanochannel. This low‐cost flexible nanochannel can also be used in various applications, including DNA sequencing and biomimetic ion channel.

## Graphical abstract

In this work, a novel low‐cost method of single nanochannel is presented based on the sacrificial electrospun polyethylene oxide (PEO) nanofiber as templates. Through this method, nanochannels of any diameter, length, and even any number of nanochannels can be prepared.

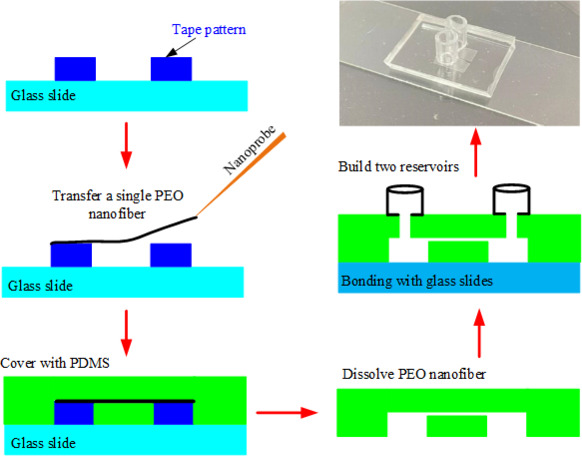

## 1 Introduction

Various recently developed nanofluidic applications of one‐dimensional nanostructures, such as nanopores[^1^, ^2^] and nanochannels,[^3^, ^4^] have shown great application prospects in energy conversion.[^5^, ^6^, ^7^, ^8^] In 2010, Kim et al. experimentally investigated power generation from silica nanochannels placed between two potassium chloride (KCl) solutions considering various combinations of concentrations; a maximum power density of 7.7 W/m^2^ was achieved.^[9]^ In 2013, Siria et al.^[10]^ described the fabrication and use of a hierarchical nanofluidic device composed of a boron nitride nanotube that pierces an ultrathin membrane and connects two fluid reservoirs. The results proved the usability of boron nitride nanotubes as membranes for osmotic power harvesting under salinity gradients, and the estimated power density surprisingly reached 4 kW/m^2^. In 2016, Feng et al. demonstrated the use of single‐layer molybdenum disulphide (MoS_2_) nanopores as osmotic nanopower generators. In their case, the maximum power density was 106 W/m^2^.^[11]^ In 2020, Van et al. reported the active control of salinity‐based power generation in nanopores using thermal and pH effects.^[12]^ Regardless of whether they are nanochannels or nanopores, most techniques to build nanofluidic devices commonly include high‐resolution fabrication steps, such as electron beam lithography,[^13^, ^14^, ^15^, ^16^] focused ion beam,[^17^, ^18^, ^19^] and transmission electron microscopy,^[20]^ which require expensive apparatus are have high operational complexity. Bellan et al. first proposed the use of electrospinning PEO nanofibers as sacrificial templates to form nanofluidic channels in PDMS.^[21]^ However, this method can only randomly prepare nanochannels and cannot accurately prepare a single controllable diameter nanochannel.

In this study, we introduced a simple and low‐cost fabrication process to build a single PDMS nanochannel. The proposed nanochannel fabrication method does not require cleanroom processes, photolithography processes, or professional etching equipment. Electrospinning was employed to generate one dimensional polyethylene oxide (PEO) nanofibres. PEO nanofibres have been widely used in electrode materials such as fuel cells and batteries.[^22^, ^23^, ^24^] Here, a single PEO nanofiber was utilised as sacrificial template to build a single PDMS nanochannel. In order to obtain a single PEO nanofiber, the PEO nanofibers are first collected on a pre prepared PDMS substrate, and then one of them is accurately transferred to the pre constructed structure using a nanoneedle tip. Using this precise transfer method, nanochannels with complex structures can be fabricated. The diameter of the nanochannel can be adjusted by the diameter of PEO nanofiber. Finally, power generation from the PDMS nanochannel was experimentally investigated by placing the nanochannel between two potassium chloride (KCl) solutions with several combinations of concentrations. These low cost nanochannels have a very broad application prospect such as DNA sequence[^25^, ^26^] and sea water desalination.[^27^, ^28^] They also can be used as template for the development of magnetic composites of new types, including the composites for biomedical applications.[^29^, ^30^, ^31^, ^32^]

## 2 Materials and Methods

**Figure 1:**
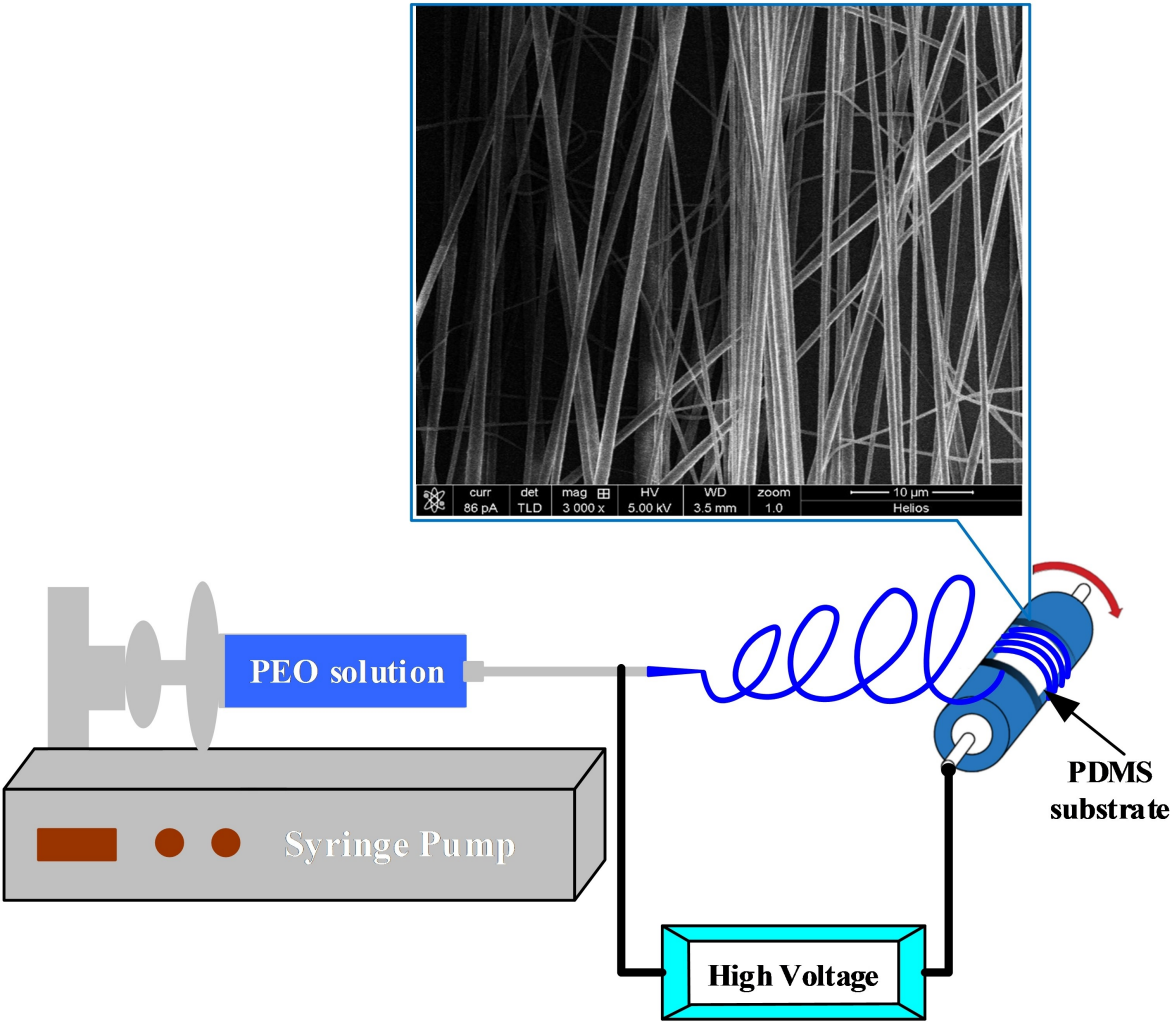
Schematic diagram of PEO nanofibers electrospinning setup. The inset picture is a high‐resolution SEM image of the PEO nanofibers.

Currently, electrospinning is a widely used technique to form nanofibers from various materials with diameters ranging from tens of nanometres to a few microns.[^21^, ^33^, ^34^] In a typical electrospinning process, a solution of polymer molecules dissolved in a solvent is supplied to a syringe needle. A high voltage is applied between the syringe needle and collector. Under the action of a high‐intensity electric field, the polymer solution forms a Taylor cone, and the jet accelerates from the Taylor cone towards the grounded collector. This jet has strong stretching flow, and as the solvent evaporates during its flight, the solvent evaporates quickly, leaving a solid fibre that is deposited on the collecting surface.^[35]^ Figure 1 shows the PEO nanofibers electrospinning setup. PEO is a water‐soluble material that is very suitable for preparing nanochannels by embedding method. As shown in Figure 1, a drum collector is used here to collect more orderly distributed nanofibers. The PDMS film (5 cm×5 cm) was pre‐attached to the drum collector. PEO powder (molecular weight of 400 000 from Sigma‐Aldrich, Inc. USA) was dissolved in deionised water at a concentration of approximately 15 wt%. The inner diameter of the flat‐tipped stainless steel needle was 0.35 mm. The applied voltage was 10 kV, and the syringe pump was set to generate a constant flow rate of 100 μL/min. The drum collector can collect PEO nanofibers arranged in a more orderly manner, so the aligned nanofibrers could be easily transferred to other substrate. The collection distance between the tip of needle and the drum collector was approximately 150 mm. The inset picture in Figure 1 is a high resolution scanning electron microscope (SEM) image of the PEO nanofiber. The diameter of the obtained PEO nanofibres ranges from 50 nm to 200 nm. The diameter could be adjusted by reducing the viscosity of the solution and increasing the applied voltage.

**Figure 2:**
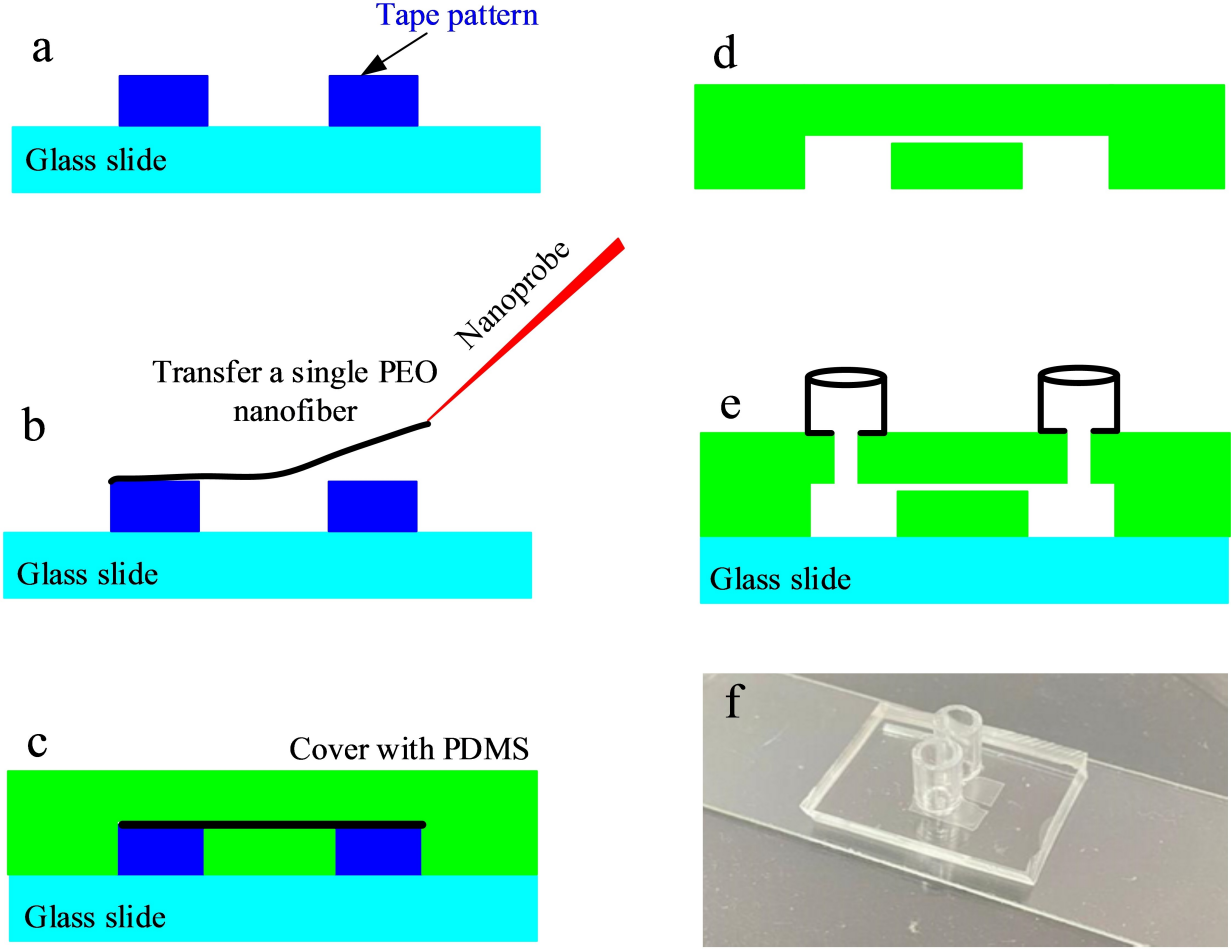
Process of the single nanochannel fabrication: a) Preparing two tape patterns on glass slide; the gap is approximately 200 μm. b) Transferring a single PEO fibre on the two patterns with the help of a home built micromanipulator. c) Covering the PEO fibre with PDMS. d) Dissolving the PEO fibre after the PDMS is solidified. e) Finally, bonding the nanochannel with glass slides and drilling two holes as solution reservoirs. f) An optical image of the obtained single PDMS nanochannel device.

To fabricate a single nanochannel, a single PEO nanofiber collected on the piece of PDMS was cut and transferred to the prepared glass slide, which was attached to two rectangular tapes,^[21]^ as shown in Figure 2a. The distance between the two tape patterns was 200 μm. After the PEO nanofiber was transferred to the top of the tape, PDMS (Sylgard 184, Dow Corning, Sigma‐Aldrich, Inc. USA) was mixed at a 1 : 8 hardener‐to‐resin ratio, after which, it was degassed and then poured over the fibre‐coated slide to be degassed again. PDMS was cured at 80 °C for 2 h. Figure 2c shows the process of pouring PDMS on the suspended nanofiber. When the PDMS was cured, a scalpel was used to release the device from the glass slide, as shown in Figure 2d. To better dissolve the PEO nanofiber, the released PDMS device was first treated with a plasma cleaner for hydrophilic treatment.^[36]^ Plasma treatment is to change the hydrophobic surface of PDMS into a hydrophilic surface, so that the water molecules could better contact and dissolve the PEO nanofibers. If the plasma hydrophilic treatment was not carried out, the PEO nanofibers would be difficult to be completely dissolved. Subsequently, the released PDMS device was soaked in water and stirred continuously with magnetic beads overnight to dissolve the PEO fibres inside the PDMS bulk. The PDMS device was then dried, and two holes were punched in it using a 3 mm flat‐tipped metal needle. In order to better bound the PDMS device with the glass slide again, both of them were exposed to an oxygen plasma for approximately 30 s. Finally, two hollow glass baths were connected above the punched holes with PDMS, as shown in Figure 2e. Figure 2f shows an optical image of the obtained PDMS nanochannel device.

**Figure 3:**
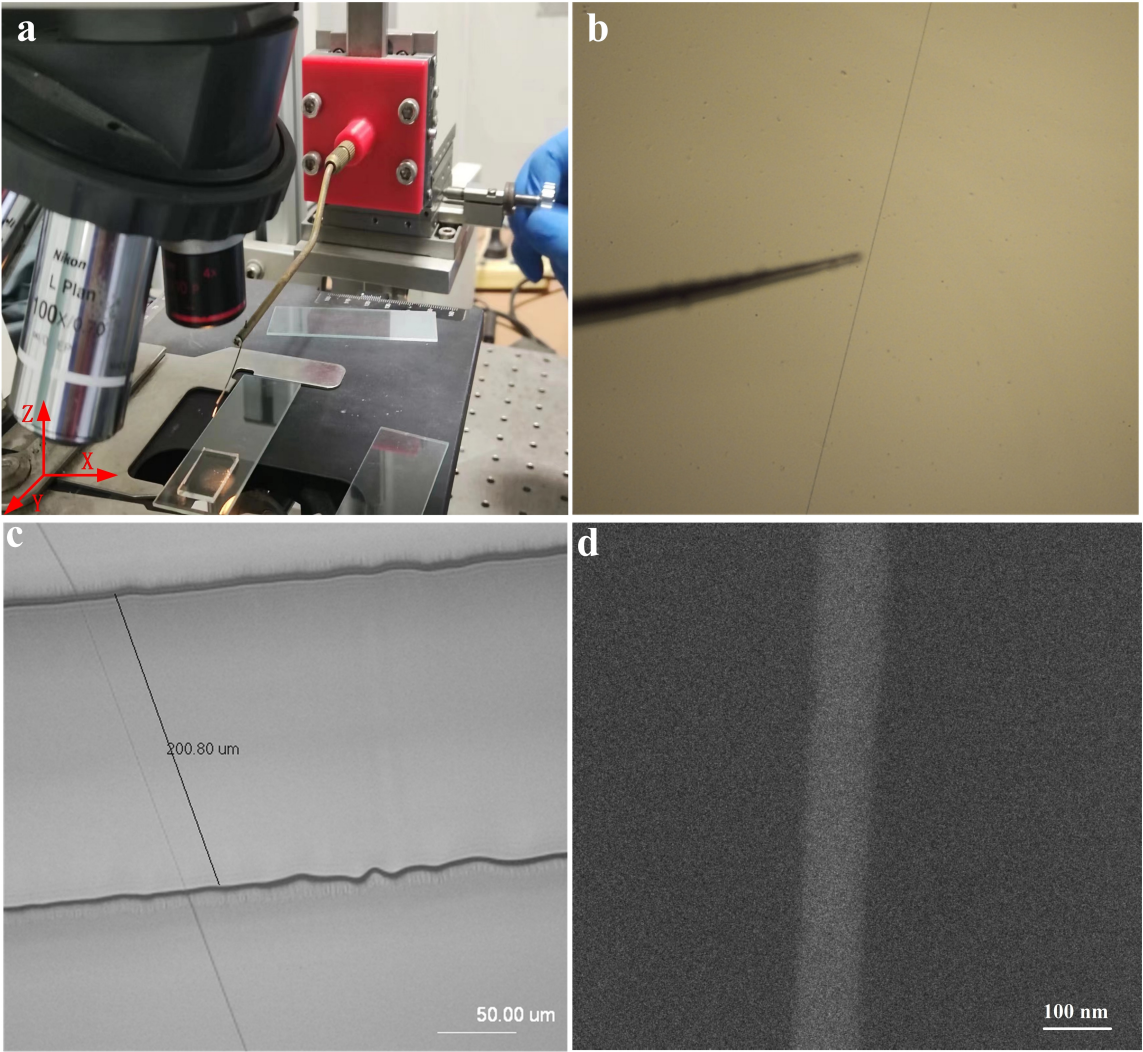
a) Photo of home built micromanipulator. b) The microscopic image of the nanoprobe and the PEO nanofiber focused together. c) A single PEO nanofiber suspended on the two tapes with a spacing of 200 μm. d) A high resolution SEM image of the suspended single PEO nanofiber.

During the fabrication process of single nanochannel, the most crucial step is the precise transfer of a single nanofiber with the help of a home built micromanipulator. Figure 3a shows the home built micromanipulator. This operating platform has 3 degrees of freedom and can achieve micrometer precision feed in X, Y, and Z directions. In the process of nanofiber transfer, the continuous nanofibers were first cut off using a nanoprobe, then lifted and transferred to the target sample. Under low magnification (10x), first locate a PEO nanofiber, and then slowly move the nanofiber near the nanoprobe, as shown in Figure 3b. After focusing the nanoprobe on the nanofiber, switch the microscope objective to 100x. Then use a nanoprobe to cut the nanofiber and lift it up. Finally, transfer the raised nanofibers to the prepared device as Figure 2b. Figure 3c was a microscope photo of a single PEO nanofiber that has been transferred. It can be seen from the figure that the length of the nanochannel is equal to the distance between two tapes, which is 200 μm. The diameter of the single PEO nanofiber was measured using SEM as shown in Figure 3d. The diameter is about 104 nm.

## 3 Results and Discussion

**Figure 4:**
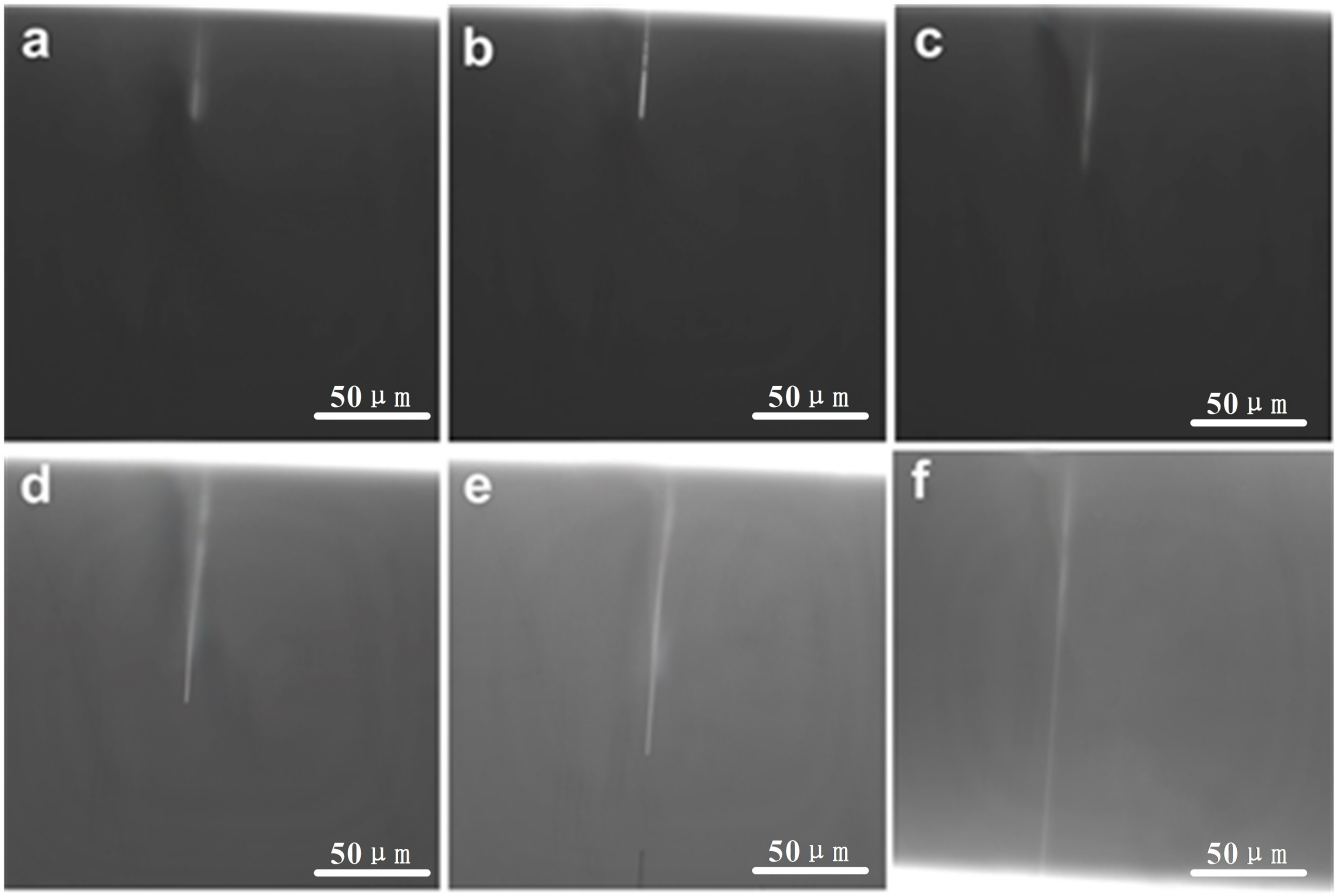
Fluorescence image of single nanochannels filling with a solution of the fluorescent dye fluorescein. Panels a‐f show the process of the fluorescein solution slowly diffusing from one side to the other side of the nanochannel.

To test the patency of nanochannels, an alcohol mixed solution of 100 μM fluorescein (Sigma‐Aldrich, USA) was filled the two reservoirs and then the nanochannel was imaged using an optical microscope in a dark field. The fluorescent solution would slowly fill the pore nanochannels, as shown in Figure 4. Figures 4a to 4 f were taken every 1 minute, and it can be seen from the figures that the fluorescence solution filling process is not uniform, which may be due to the uneven inner diameter of PDMS nanochannels. Complete dissolution of PEO nanofibres is a prerequisite for ensuring the repeatability of subsequent experiments. If the PEO nanofibres are incompletely or partially dissolved, the fluorescence solution in the channel is discontinuous and the value of conductivity in subsequent measurements will be almost zero. Compared with multiple nanochannels, single nanochannel do not need to consider the average pore size effect of multiple channels and can directly observe changes in pore properties.

**Figure 5:**
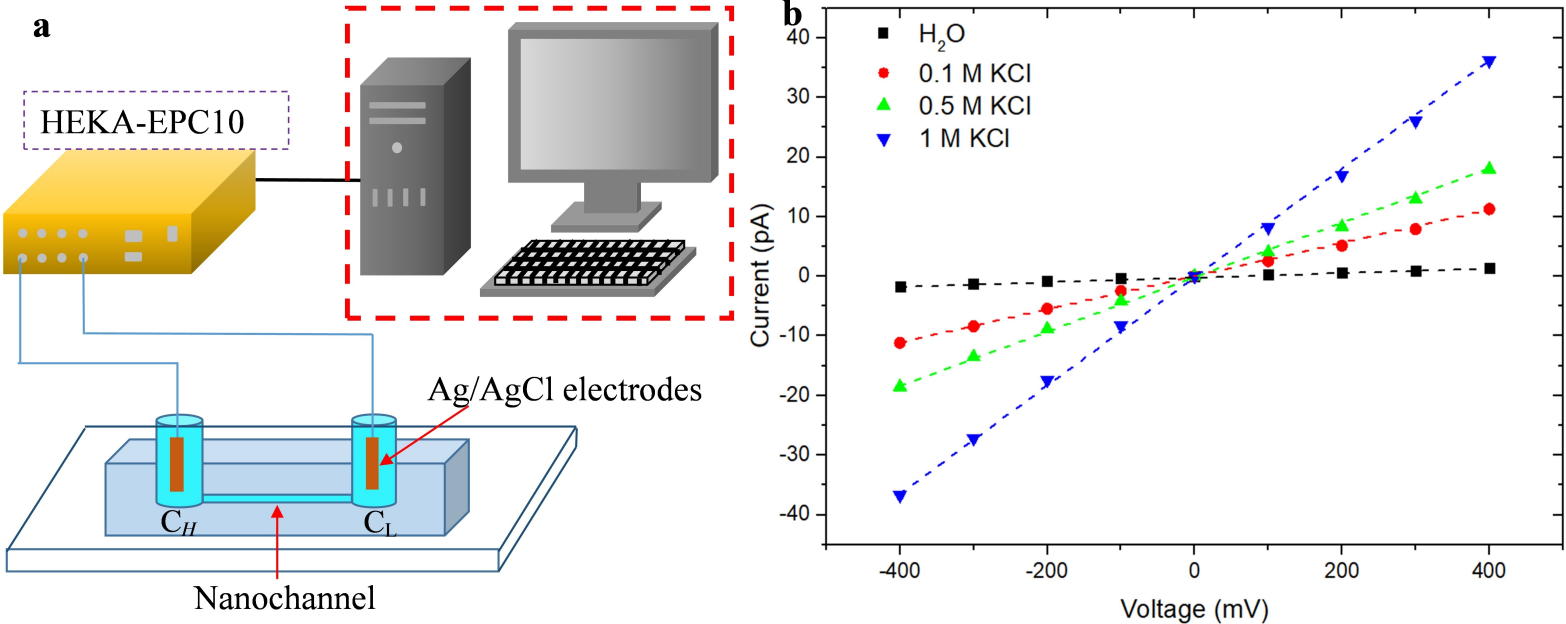
Experimental setup for nanochannel conductivity measurement: a) Schematic of the ionic current measurement. b) *I–V* curves of the PDMS nanochannel at different KCl concentrations.

Due to the difficulty in directly characterizing the diameter of PDMS nanochannels using electron microscopy, their effective diameter can only be estimated through ion current measurements. Figure 5a was the schematic diagram of the conductivity characteristics measured by the single nanochannel. Before measuring the ionic conductivity of the PDMS nanochannel, it was first necessary to perform hydrophilic treatment on the nanochannels with a plasma cleaner. Hydrophilic treatment can ensure the continuity of fluid in the nanochannel. Subsequently, the two reservoirs were filled with degassed, filtered KCl electrolyte, and Ag/AgCl electrodes were immersed from both sides of the nanochannel. The ionic current of the nanochannnel was tested using a patch clamp amplifier with pico‐ampere sensitivity (HEKA EPC 10 USB, HEKA Instruments, USA). The setup was placed in a double Faraday cage to block the electrical noise from the environment. Similarly, we vacuumed the solution before each experiment to ensure its repeatability by avoiding errors caused by air bubbles.

Figure 5b shows the *I–V* curve of the PDMS nanochannel at various KCl concentrations. This curve was obtained by sweeping the applied bias from −400 mV to 400 mV in steps of 100 mV and recording the corresponding ionic current at each point. All the *I–V* curve experiments were performed at room temperature, and the measured pH of the DI water and all other salt solutions was ~5. The ion conductance of the PDMS nanochannel was extracted by fitting the slope of the distinct linear *I–V* curve as the dash lines show in Figure 5b. After obtaining the ionic conductance, the diameter of the nanochannel can be calculated according to the following formula:[^37^, ^38^]

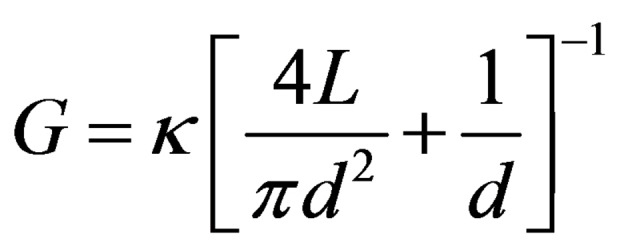




where *G* is the measured conductance of the PDMS nanochannel, *d* is the diameter of the nanochannel, *L* is the length of the nanochannel, and *κ* represents the bulk ionic conductivity of KCl. The length of the nanochannel was approximately 200 μm, and the *κ* of 1 M KCl is 9.83 S/m.^[39]^ According to Eq. (1), the calculated diameter of the PDMS nanochannel is ~49 nm, which is much smaller than the original diameter of the PEO nanofiber (~104 nm). This may be caused by three reasons: first, the uneven diameter of PEO nanofibers; second, the compression of PDMS during the curing process leading to a decrease in the size of PEO nanofibers; and third residual PEO in the nanochannel.

**Figure 6:**
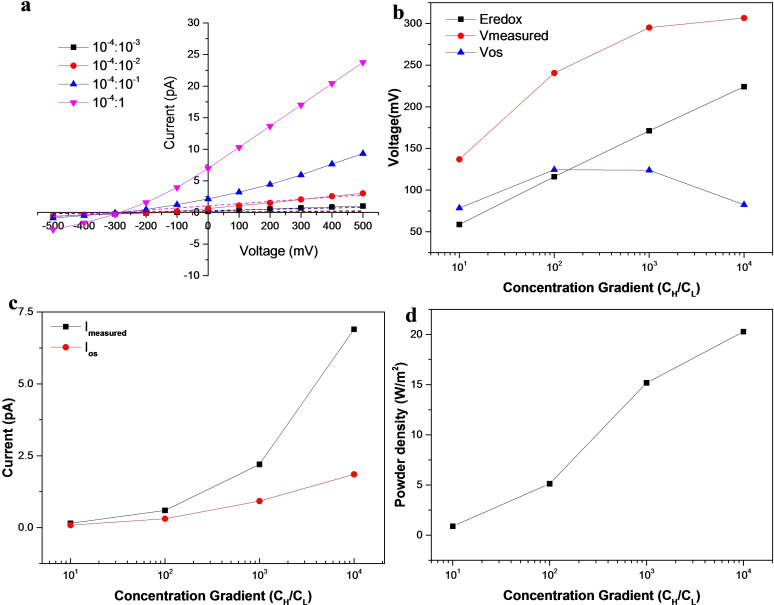
Single PDMS nanochannel power generator. a) *I–V* curve for different KCl concentration gradients. b) The osmotic voltage generated from the single PDMS nanochannel with various concentration gradient. c) The osmotic current generated from the single PDMS nanochannel. d) Dependence of the maximum power output density on the concentration gradient of the single PDMS nanochannel .

To investigate the performance of the single PDMS nanochannel for harvesting salinity gradient power, different concentration gradients of the KCl solution were tested separately. The low concentration at the cis reservoir (C_*L*_) was fixed at 10^−4^ M, while the high concentration at the trans reservoir (C_*H*_) was gradually increased from10^−3^ M to 1 M, with the concentration gradient ratio defined as C_*H*_ */*C_*L*_. As shown in Figure 6a, a large osmotic current and osmotic potential are obtained using a single PDMS nanochannel.

The current measured including the osmotic current generated by the nanochannel generator and the contribution of the electrode redox reaction. The osmotic current (*I*_os_) and osmotic voltage (*V*_os_) can be expressed as:^[11]^

(2) $$V_{os}=V_{measured}-E_{redox}$$

(3) $$I_{os}=\left(V_{os}/V_{measured}\right)\times I_{measured}$$

As shown in Figure 6b, when concentration gradient (C_*H*_ */*C_*L*_) increased from 10 to 10 000‐fold, *V*_os_ experienced a peak value at a medium concentration gradient of about 100‐fold. In theory, the higher concentration gradient should generate higher *V*_os_. However, the high concentration reduced the Debye length and led to a decrease in the ion selectivity of the nanochannel. When the concentration gradient reaches 100 times, it ultimately leads to a decrease in *V*_os_.^[40]^ The osmotic current increased as the concentration gradient increased as show in Figure 6c indicated that higher concentration gradient can release more Gibbs free energy. The maximum output power density of the nanochannel, P_*out*_, as a function of concentration gradient (C_*H*_ */*C_*L*_); P_*out*_ is defined by^[40]^ 4

(4) $$P_{out}=\frac{P_{\max}}{\pi d^{2}}=\frac{V_{os}I_{os}}{\pi d^{2}}$$

From Figure 6d, the maximum power output density attained in the 200 μm length PDMS nanochannel was high to 20 W/m^2^. Although this energy conversion density was much lower than the research results of Feng et al.^[11]^ The main reason was that the length of our nanochannels was at the micrometer level, while the thickness of the molybdenum disulfide film reported by Feng Jiandong is only a few nanometers. However, in the study by Zhang Yang et al,^[40]^ the output power density can reached 705 W/m2. They ascribed the enhancement of energy harvesting to the appropriate length of nanochannel that provides a good balance between the desirable ion selectivity and the unfavorable large resistance from the nanochannel. So if the length of our channel is further shortened to the optimal length, it is believed that the output power density would be higher.

## 4 Conclusions

In summary, a novel low‐cost method to fabricate single PDMS nanochannels was developed based on sacrificial electrospun PEO nanofibre as templates. A single PEO nanaofiber was collected on the piece of PDMS and then it was cut and transferred to the prepared glass slide by a miniprobe. Afterwards, a single nanochannel could be formed by embedding the PEO nanofiber and finally dissolving it. The diameter and length of the nanochannel could be controlled by adjusting the size of the nanofiber. This nanofabrication technique allowed for the simple construction of integrated micro‐ and nano‐fluidic PDMS structures without lithographic nanofabrication techniques. This method could also prepare any number of nanochannels, not just nanosingle channels. According to the conductance calculation, the diameter of the nanochannel was much smaller than the original size of the nanofiber. Additionally, we experimentally investigated the power generation from nanochannel placed between two KCl solutions with various concentration gradients. The results showed that the maximum value of the estimated power density reaches approximately 20 W/m^2^. Although this energy conversion density was not the highest, it was still higher than the power density reported in most literature indicating that low‐cost PDMS nanochannels have great potential for achieving an accurate power supply for ultra‐low‐power devices.

## Conflict of interests

The authors declare no conflict of interest.

5

## Data Availability

The data that support the findings of this study are available in the supplementary material of this article.

## References

[1] Z. Huang , Y. Zhang , T. Hayashida , Z. W. Ji , Y. H. He , M. Tsutsui , X. S. Miao , M. Taniguchi , Appl. Phys. Lett. 2017, 111(26), 263104.

[2] M. Tsutsui , K. Yokota , I. W. Leong , Y. He , T. Kawai , Cell Rep. Phys. Sci. 2022, 3(10), 101065.

[3] K. Yazda , K. Bleau , Y. N. Zhang , X. Capaldi , T. St-Denis , P. Grutter , W. W. Reisner , Nano Lett. 2021, 21(10), 4152–4159. 33982572 10.1021/acs.nanolett.0c04704

[4] D. K. Kim , C. H. Duan , Y. F. Chen , A. Majumdar , Microfluid. Nanofluid. 2010, 9(6), 1215–1224.

[5] A. Soozanipour , H. Sohrabi , F. Abazar , A. Khataee , A. Noorbakhsh , M. Asadnia , A. Taheri-Kafrani , M. R. Majidi , A. Razmjou , Adv. Mater. Technol. 2021, 6(10), 2000765.

[6] X. Hou , W. Guo , L. Jiang , Chem. Soc. Rev. 2011, 40(5), 2385–2401. 21308139 10.1039/c0cs00053a

[7] J. H. Lu , Y. A. JiangA , P. Yu , W. Jiang , L. Q. Mao , Chem. Asian J. 2022, 17(10), e202200158. 35324076 10.1002/asia.202200158

[8] D. F. Ding , P. C. Gao , Q. Ma , D. G. Wang , F. Xia , Small 2019, 15(32), 1804878. 10.1002/smll.201804878 30756522

[9] D.-K. Kim , C. Duan , Y.-F. Chen , A. Majumdar , Microfluid. Nanofluid. 2010, 9(6), 1215–1224.

[10] A. Siria , P. Poncharal , A. L. Biance , R. Fulcrand , X. Blase , S. T. Purcell , L. Bocquet , Nature 2013, 494(7438), 455–458. 23446417 10.1038/nature11876

[11] F. D. Feng , M. Graf , K. Liu , D. Ovchinnikov , D. Dumcenco , M. Heiranian , V. Nandigana , N. R. Aluru , A. Kis , A. Radenovic , Nature 2016, 536(7615), 197–200. 27409806 10.1038/nature18593

[12] V. P. Mai , R. J. Yang , RSC Adv. 2020, 10(32), 18624–18631. 35518343 10.1039/d0ra02329a PMC9053878

[13] S. H. Park , H. J. Shin , Y. H. Kim , D. Y. Yang , J. C. Lee , S. Lee , J. Micromech. Microeng. 2012, 22(9), 095019.

[14] C. Danelon, C. Santschi, J. Brugger, H. J. L. Vogel, *Langmuir* **2007**, *22(25)*, 10711–10715. 10.1021/la061321c 17129050

[15] Mirwais Aktary, Martin O. Jensen, Kenneth L. Westra, Michael J. Brett, Mark R. Freeman, Journal of Vacuum Science & Technology B: Microelectronics and Nanometer Structures Processing, Measurement, and Phenomena, 21(4), L5-L7.

[16] C. Wu , T. G. Lin , Z. K. Zhan , Y. Li , S. C. H. Tung , W. C. Tang , W. J. Li , Microsyst. Nanoeng. 2017, 3(1), 1–9. 10.1038/micronano.2016.84 PMC6445022 31057852

[17] K. B. Kim , G. Hobler , A. Steiger , A. Lugstein , E. Bertagnolli , E. Platzgummer , H. Loeschner , Int. J. Precis. Eng. Man. 2011, 12(5), 893–898.

[18] J. Li , D. Stein , C. McMullan , D. Branton , M. J. Aziz , J. A. Golovchenko , Nature 2001, 412(6843), 166–169. 11449268 10.1038/35084037

[19] L. D. Menard , J. M. Ramsey , Nano Lett. 2011, 11(2), 512–517. 21171628 10.1021/nl103369g PMC3125600

[20] T. Deng , M. W. Li , Y. F. Wang , Z. W. Liu , Sci. Bull. 2015, 60(3), 304–319.

[21] L. M. Bellan , E. A. Strychalski , H. G. Craighead , J. Vac. Sci. Technol. B 2008, 26(5), 1728–1731.

[22] M. Thomas , S. Rajiv , Electrochim. Acta 2020, 341, 136040.

[23] H. G. Li , Y. F. Ho , M. M. M. Ahmed , H. C. Liang , S. W. Kuo , Chem. Eur. J. 2019, 25(44), 10456–10463. 31206853 10.1002/chem.201901724

[24] G. Shanmgam , V. Mathew , B. Selvaraj , P. M. Thanikachalam , J. Kim , M. Pichai , A. Natarajan , A. I. Almansour , ChemistrySelect 2022, 7(1), e202103007.

[25] P. Fanzio , V. Mussi , C. Manneschi , E. Angeli , G. Firpo , L. Repetto , U. Valbusa , Lab Chip 2011, 11(17), 2961–2966. 21750811 10.1039/c1lc20243j

[26] S. K. Min , W. Y. Kim , Y. Cho , K. S. Kim , Nat. Nanotechnol. 2011, 6(3), 162–165. 21297626 10.1038/nnano.2010.283

[27] M. Heiranian , A. B. Farimani , N. R. Aluru , Nat. Commun. 2015, 6, 8616. 26465062 10.1038/ncomms9616 PMC4634321

[28] C. C. Lai , C. J. Chang , Y. S. Huang , W. C. Chang , F. G. Tseng , Y. L. Chueh , Nano Energy 2015, 12, 394–400.

[29] D. Ling , W. Park , S. J. Park , Y. Lu , K. S. Kim , M. J. Hackett , B. H. Kim , H. Yim , Y. S. Jeon , K. Na , T. Hyeon , J. Am. Chem. Soc. 2014, 136(15), 5647–5655. 24689550 10.1021/ja4108287

[30] K. J. Lodewijk , E. Fernandez , A. Garcia-Arribas , G. V. Kurlyandskaya , V. N. Lepalovskij , A. P. Safronov , B. J. Kooi , J. Appl. Phys. 2014, 115(17).

[31] G. V. Kurlyandskaya , A. P. Safronov , T. V. Terzian , N. S. Volodina , I. V. Beketov , L. Lezama , L. M. Prieto , Ieee Magn. Lett. 2015, 6, 1–4.

[32] S. Tanaka , Y. V. Kaneti , N. L. W. Septiani , S. X. Dou , Y. Bando , M. S. A. Hossain , J. Kim , Y. Yamauchi , Small Methods 2019, 3(5), 1800512.

[33] X. Wang , G. F. Zheng , L. Xu , W. Cheng , B. L. Xu , Y. F. Huang , D. H. Sun , Appl. Phys. A Mater. 2012, 108(4), 825–828.

[34] M. Wang , N. Jing , C. B. Su , J. Kameoka , C. K. Chou , M. C. Hung , K. A. Chang , Appl. Phys. Lett. 2006, 88(3).

[35] Z. M. Huang , Y. Z. Zhang , M. Kotaki , S. Ramakrishna , Compos. Sci. Technol. 2003, 63(15), 2223–2253.

[36] J. Ma , Q. Y. Zeng , L. J. Zhan , J. W. Mo , Y. Zhang , Z. H. Ni , NANO 2020, 15(11), 2050148.

[37] M. Wanunu , T. Dadosh , V. Ray , J. M. Jin , L. McReynolds , M. Drndic , Nat. Nanotechnol. 2010, 5(11), 807–814. 20972437 10.1038/nnano.2010.202

[38] J. Ma , K. Li , Z. W. Li , Y. H. Qiu , W. Si , Y. Y. Ge , J. J. Sha , L. Liu , X. Xie , H. Yi , Z. H. Ni , D. Y. Li , Y. F. Chen , J. Am. Chem. Soc. 2019, 141(10), 4264–4272. 30773010 10.1021/jacs.8b08488 PMC6675019

[39] F. Macdonald , D. R. Lide , Abstr. Pap. Am. Chem. Soc. 2003, 225, U552–U552.

[40] Y. Zhang , Z. Huang , Y. H. He , X. S. Miao , Nanotechnology 2019, 30(29), 295402. 30861495 10.1088/1361-6528/ab0ed8

